# Endocardite Infecciosa: Novos Espectros, a Mesma Gravidade

**DOI:** 10.36660/abc.20230117

**Published:** 2023-03-27

**Affiliations:** 1 Faculdade de Medicina da Universidade de São Paulo Instituto do Coração – InCor São Paulo SP Brasil Instituto do Coração – InCor - Faculdade de Medicina da Universidade de São Paulo, São Paulo, SP – Brasil

**Keywords:** Endocardite Infecciosa, Cirurgia Cardíaca, Doença das Valvas Cardíacas

A endocardite infecciosa é uma doença rara, mas com elevada taxa de complicações graves e que muitas vezes pode ser encarada como uma síndrome.^1^ Uma avaliação fria dos dados publicados internacionalmente^2^ nos mostra que apesar de todo o avanço tecnológico e uma tendência precoce a intervenção cirúrgica, não houve uma grande redução nos desfechos clínicos, principalmente mortalidade, mas uma busca mais detalhada de informações pode nos trazer outros pontos de vista.

O artigo de Jorge MS et al.,^3^ corrobora a ideia de diagnósticos e intervenções precoces nesse amplo espectro de pacientes e observar a mudança epidemiológica dos pacientes ao longo dos anos é chave fundamental para uma leitura ampla do manejo da endocardite infecciosa contemporânea.^4^

Embora a patologia da endocardite seja a mesma, a doença em si surge como um antigo problema, mas com roupagem completamente diferente. Na era dos primeiros antimicrobianos disponíveis, os pacientes eram jovens e apresentavam relativamente poucas comorbidades, como cardiopatias reumática ou congênita. Recentemente, em um momento de antimicrobianos de amplo espectro e potencial bactericida mais robusto, o paciente que se apresenta tem outro perfil, sendo mais idoso, com múltiplas comorbidades e clinicamente mais instável.^2^

Ao observar o comportamento evolutivo dos pacientes analisados, Jorge MS et al.,^3^ também destaca a mudança do perfil clínico epidemiológico e das cepas microbiológicas envolvidas, situação que está intimamente relacionada ao perfil do paciente, muitas vezes institucionalizado. O que antes tinha como um grande protagonista o Streptococcus, os dados contemporâneos apontam para a maior prevalência do Staphylococcus como agente causador da endocardite infecciosa.^5^

Embora tenha sido, até o momento, pouco abordado de forma ampla a irrestrita, as mudanças no manejo da profilaxia antibiótica para prevenção da endocardite infecciosa também podem ter impactado essa evolução ao longo dos últimos 30 anos, em que cada vez mais temos uma conduta conservadora nas diretrizes internacionais, contrastando com as orientações encontradas na última diretriz nacional de valvopatias.^6,7^

Nesse contexto, um diagnóstico rápido e preciso é o primeiro passo para oferecer ao paciente a chance de uma intervenção mais assertiva e no momento certo. Um diagnóstico tardio e a procrastinação de uma terapia antimicrobiana adequada levam a complicações e piores resultados clínicos.^8^

A apresentação clínica é diversa, partindo desde quadros sépticos graves até síndromes de febre de origem indeterminada ou mesmo manifestações puramente cardiovasculares como insuficiência cardíaca.^2^ O autor^3^ inclusive aponta para a insuficiência cardíaca congestiva como principal comorbidade associada aos pacientes que foram acompanhados, o que pode trazer uma confusão clínica e um desafio diagnóstico ainda mais complexo, visto que a presença dessa manifestação pode atrasar um diagnóstico preciso que necessitará, mais do que antes, de outros critérios de Duke, como visto nos métodos de imagem.^9-11^

Os desafios contemporâneos da Endocardite Infecciosa são diversos e a prevenção é sem sombra de dúvidas a melhor estratégia a ser empregada.^12^ Uma vez diante da possibilidade, o rápido diagnóstico e uma terapia individualizada parecem ser a melhor estratégia para reduzir as complicações, sendo o tratamento cirúrgico, um procedimento que tem papel crescente e decisivo em grupos de pacientes mais graves. A busca por informações desses pacientes proporcionará à cardiologia a chance de transformar os desafios em caminhos pavimentados para as melhores respostas terapêuticas na endocardite infecciosa.
