# Supplementary material for: Endocardite Infecciosa: Novos Espectros, a Mesma Gravidade
Source: Arq Bras Cardiol. 2023 Mar 27;120(3):e20230117. [Article in Portuguese] doi: 10.36660/abc.20230117 (PMC10263453; doi:10.36660/abc.20230117)
Supplement: Translation

# Translation: Infective Endocarditis: New Spectra, Same Severity

**Affiliations:** 1 São Paulo SP Brazil Instituto do Coração – InCor - Faculdade de Medicina da Universidade de São Paulo, São Paulo, SP – Brazil

**Keywords:** Infective Endocarditis, Cardiac Surgery, Heart Valve Disease

Infective endocarditis is a rare disease with a high rate of serious complications and can often be seen as a syndrome.^1^ A cold assessment of data published internationally^2^ shows us that despite all the technological advances and an early trend towards surgical intervention, there was no great reduction in clinical outcomes, especially mortality, but a more detailed search for information may bring us other points of view.

The article by Jorge MS et al.^3^ corroborates the idea of early diagnoses and interventions in this broad spectrum of patients, and observing the epidemiological change of patients over the years is a fundamental key to a broad reading of the management of contemporary infective endocarditis.^4^

Although the pathology of endocarditis is the same, the disease appears as an old problem in a completely different guise. In the era of the first antimicrobials available, patients were young and had relatively few comorbidities, such as rheumatic or congenital heart disease. Recently, in a time of broad-spectrum antimicrobials and more robust bactericidal potential, the patient who presents has a different profile, is older, has multiple comorbidities, and is clinically more unstable.^2^

By observing the evolutionary behavior of the analyzed patients, Jorge MS et al.^3^ also highlight the change in the epidemiological, clinical profile, and microbiological strains involved. This situation is closely related to the profile of the patient, who is often institutionalized. What used to have Streptococcus as a major protagonist, the contemporary data point to a higher prevalence of Staphylococcus as the causative agent of infective endocarditis.^5^

Although it has so far been little discussed in a broad or unrestricted way, the changes in the management of antibiotic prophylaxis for the prevention of infective endocarditis may also have impacted this evolution over the last 30 years, in which we have an increasingly conservative approach in international guidelines, contrasting with the guidelines found in the last national guideline on valve diseases.^6,7^

In this context, a quick and accurate diagnosis is the first step to offer the patient the chance of a more assertive and timely intervention. A late diagnosis and the procrastination of adequate antimicrobial therapy lead to complications and worse clinical outcomes.^8^

The clinical presentation is diverse, ranging from severe sepsis to fever syndromes of an undetermined origin or even purely cardiovascular manifestations such as heart failure.^2^ The author^3^ even points to congestive heart failure as the main comorbidity associated with patients who were followed up, which can lead to clinical confusion and an even more complex diagnostic challenge since the presence of this manifestation can delay an accurate diagnosis that will require more than before, of other Duke criteria, as seen on imaging methods.^9-11^

The contemporary challenges of Infective Endocarditis are diverse, and prevention is undoubtedly the best strategy to be employed.^12^ Once faced with the possibility, rapid diagnosis, and individualized therapy seem to be the best strategy to reduce complications, with treatment surgery, a procedure that plays a growing and decisive role in groups of more severe patients. The search for information from these patients will allow cardiology to transform challenges into paved paths for the best therapeutic responses in infective endocarditis.
